# Flow diversion effect of the leo braided stent for aneurysms in the posterior and distal anterior circulations: A multicenter cohort study

**DOI:** 10.3389/fneur.2022.957709

**Published:** 2022-09-27

**Authors:** Yu Duan, Binbin Xu, Xuanfeng Qin, Renling Mao, Yuanyuan Hu, Bin Zhou, Jian Li, Gong Chen

**Affiliations:** ^1^Departments of Neurosurgery, Huadong Hospital, Fudan University, Shanghai, China; ^2^Departments of Neurosurgery, Shanghai Putuo District People′s Hospital, Shanghai, China; ^3^Departments of Neurosurgery, Huashan Hospital, Fudan University, Shanghai, China

**Keywords:** endovascular treatment, flow diversion, aneurysm, stents, multicenter study

## Abstract

**Background and purpose:**

The treatment of aneurysms located in the posterior and distal anterior circulations remains a challenge. Leo stents with a flow diversion (FD) effect may be a potential option, which needs to be clearly studied.

**Methods:**

From January 2016 to October 2021, 133 patients with 145 aneurysms in the posterior and distal anterior circulations, treated with Leo stents, were retrospectively analyzed in three neurosurgical centers. Data on demographic information, aneurysm characteristics, procedural outcomes, postoperative course, and aneurysm occlusion were retrospectively analyzed.

**Results:**

After immediate surgery, 90 aneurysms (60.1%) were in complete occlusion [Raymond-Ray Occlusion Class (RROC) 1 and O'Kelly Marotta (OKM) grade D], 29 aneurysms (20%) in good occlusion (RROC 2 and OKM grade C), 17.9% in incomplete occlusion (RROC 3a or OKM grade B), and no aneurysms in invalid occlusion (RROC 3b and OKM grade A). A total of 112 patients with 117 aneurysms received angiographic follow-up (mean 11.4 months), and the degree of occlusion showed a significant improvement (Z = 3.900, *p* < 0.001). The complete occlusion rate increased to 84.6% (99/117), while good and incomplete occlusion decreased to 6.8% (8/117) and 8.6% (10/117), respectively. A total of 14 cases (10.5%) presented narrowing of the parent artery, and nine cases (6.8%) had injured side branches. Cerebral hemorrhage occurred in four patients (3.0%), and symptomatic ischemic infarction occurred in six patients (4.5%). The final permanent morbidity (mCS ≥3) and mortality were 2.8% (3/133) and 0.8% (1/133), respectively. For 82 aneurysms treated by stent-assisted with coiling (SAC), large-sized, ruptured aneurysms (χ^2^ = 7.767, *p* = 0.005) occurred. For 63 aneurysms treated by LEO stent monotherapy (LSM), multiple aneurysms, fusiform aneurysms (χ^2^ = 18.958, *p* < 0.01), and/or small-sized aneurysms (*Z* = −2.692, *p* = 0.007) occurred.

**Conclusions:**

Leo stents are safe and effective for aneurysms located in the posterior and distal anterior circulations. The overall degree of occlusion improved during a follow-up because of the FD effect of Leo stents. Aneurysms in these areas should be treated with personalized measures.

## Highlights

- About 133 patients with 145 aneurysms in the posterior and distal anterior circulations were retrospectively analyzed in multi-neurosurgical centers.
- The safety and efficacy of Leo stents were verified.
- The FD effect of Leo stents had improved aneurysm occlusion during a follow-up.
- Aneurysms with different characteristics should be treated with personalized measures.

## Introduction

Endovascular treatment (EVT) of aneurysms located in the posterior and distal anterior circulations remains challenging and involves a high risk of regrowth, aneurysm rupture, and parent artery occlusion, leading to disastrous consequences, due to small, tortuous parent arteries and the vital blood supply areas (1, 2).

As self-expanding stents, the metal coverage of Leo plus and Leo plus baby is ~14 and 18%, respectively, and between laser-cut stents and flow-diverting stents (3). Leo stents have been proven to have a flow diversion (FD) effect. In 2005, the Leo self-expanding stent was applied for the first time to treat a wide-neck intracranial aneurysm, with the advantage of greater artificial coverage of the aneurysm neck (4). Soon afterwards, Leo stents have been attempted as monotherapy with stent or stent-assisted with coiling (SAC), for complicated aneurysms in some tortuous segments (5, 6).

The flow velocity in the aneurysm and the wall shear stress are two important hemodynamic parameters associated with the growth and rupture of intracranial aneurysms (7, 8). Leo stent implantation produces hemodynamic and biological effects on the parent artery to promote aneurysm occlusion, which cannot only redirect blood flow and decrease wall stress in the aneurysm, but also induce neointimal proliferation in the parent artery (5, 9).

However, the FD effect of Leo stents still needs to be clearly studied, especially for those aneurysms located in the posterior and distal anterior circulations. We collected a retrospective series of 133 patients with 145 aneurysms covered by LEO stents at three neurosurgical centers and focused on the following questions: (1) the safety and clinical efficacy of Leo for aneurysms located in the posterior and distal anterior circulations; (2) remodeling effect of Leo stents; and (3) clinical characteristic between LEO stent monotherapy (LSM) and SAC.

## Materials and methods

### Study design

From January 2016 to October 2021, patients with aneurysms treated with Leo plus or Leo plus baby stent were consecutively enrolled and retrospectively analyzed at our three neurosurgical centers. Inclusion criteria were as follows: (1) patients diagnosed with intracranial aneurysm(s) by digital subtraction angiography (DSA); (2) patients more than 14 years old; and (3) deployment *via* Leo plus or Leo baby plus stent(s). Exclusion criteria were as follows: (1) presence of aneurysms located in the internal carotid artery segment; (2) deployment by other kinds of stents; and (3) loss to follow-up. Data on demographic information, aneurysm characteristics, procedural outcomes, postoperative course, and aneurysm occlusion were analyzed.

### Perioperative drug management

For patients with unruptured aneurysm, aspirin (100 mg/day) and clopidogrel (75 mg/day) were administered for at least 3 days before the operation. For patients with acute subarachnoid hemorrhage, 300 mg of aspirin and 150 mg of clopidogrel were taken 4 h before EVT. Leo plus or Leo plus baby was deployed under intraoperative systemic heparinization (80 U/kg by intravenous injection at first and then 1,000 U/h). Aspirin (100 mg/day) and clopidogrel (75 mg/day) were continued for at least 3 months after EVT, and then aspirin (100 mg/day) was administered separately for at least 2 years or for life, according to the result of angiographic follow-up.

### Endovascular therapy

Endovascular treatment was performed on the angiographic system (Artis zeego, Siemens AG, Healthineers, Forchheim, Germany). After general anesthesia, a long femoral sheath and a 6F guiding catheter (Johnson & Johnson, NJ, USA) were deployed, then the microcatheter was introduced at the target location. Treatment planning was carefully determined by three-dimensional (3D) rotational angiography.

Leo plus (Balt Extrusion, Montmorency, France) has its own microcatheter, and the Leo plus baby stent was delivered *via* a 0.017″ microcatheter or Echelon 10 (Medtronic, Irvine, CA, USA). Tirofiban hydrochloride (Yuanda Pharmaceutical Co. LTD, China) was used for acute thrombus within the stent, and if the stent was poorly opened, microcatheter massage of the stent or balloon dilatation was considered. Intracranial hemorrhage was routinely ruled out after the procedure by flat detector computed tomography (FD-CT).

### Clinical follow-up

The first angiographic follow-up was usually scheduled at 6 months or more after surgery. Without special conditions, magnetic resonance angiography (MRA) without contrast medium injection was suggested once a year. The occlusion status of aneurysms treated with LSM and SAC was evaluated using the O'Kelly Marotta (OKM) grading scale (10) and the Raymond-Ray Occlusion Class (RROC) (11), respectively. OKM grade D and RROC 1 were defined as complete occlusion, OKM grade C and RROC 2 as good occlusion, OKM grade B and RROC 3a as incomplete occlusion, and OKM grade B and RROC3b as invalid occlusion. Clinical neurological function was assessed using the modified Rankin scale (mRS).

### Statistical analysis

Data were analyzed with the SPSS23.0 statistical software package (SPSS, Chicago, IL, USA). Normally distributed measurement data [shown as mean and standard deviation (SD)] were tested by *t*-test. Non-normally distributed measurement data (shown as median and quartile spacing) were tested using the rank sum test (Mann–Whitney *U*-test]. Categorical variables (shown as number and percentage) were tested using Fisher's exact test to compare rates between groups. Complication risks were analyzed using multivariate logistic regression analysis. A *p*-value of < 0.05 was considered statistically significant.

## Results

### Demographic information

A total of 133 patients with 145 aneurysms were enrolled in our study (Table 1). There were 80 women and 53 men with an average age of 54.0 ± 12.1 years (19–76 years). Of 145 aneurysms, 86 (59.3%) were in the posterior circulation and 59 (40.7%) were in the distal anterior circulation; and 67 (47.6%) were saccular aneurysms and 76 (52.4%) were fusiform aneurysms. A total of 21 patients had a subarachnoid hemorrhage before treatment. Of these, 19 (90.5%) were mild (Hunt–Hess grades 1–2).

**Table 1:** Characteristic of 133 patients.

**Clinical information**	**Date (*n* = 133)**
Age (years old)	54.0 ± 12.1
Female (*n*, %)	80, 60.2%
**Cardiovascular risk factors (** ***n*** **, %)**	
Smoking	4, 0.3%
Drinker	5, 0.38%
Hypertension	17, 12.8%
Diabetes	12, 9.0%
SAH	26, 19.5%
Cerebral infarction	6, 4.5%
**Hunt-hess grade**	
1	10, 7.5%
2	9, 6.8%
3	2, 1.5%
**Number of aneurysms in a patient**	
1	122, 91.7%
2	10, 7.5%
3	1, 0.8%
**Endovascular methods**	
LSM	51, 38.3%
SAC	82, 61.7%

SAH, subarachnoid hemorrhage; LSM, Leo stent monotherapy; SAC, stent-assisted with coiling.

### Angiographic outcomes

As shown by the immediate occlusion results, 90 aneurysms (60.1%) were in complete occlusion (RROC 1 and OKM grade D), 29 aneurysms (20%) in good occlusion (RROC 2 and OKM grade C), 26 (17.9%) in incomplete occlusion (RROC 3a or OKM grade B), and no aneurysms in invalid occlusion (RROC 3b and OKM grade A). After a mean of 11.4 months (6 to 17 months), 112 patients with 117 aneurysms received angiographic follow-up and the degree of occlusion improved significantly (*Z* = −3.900, *p* < 0.001). The rate of complete occlusion increased to 84.6% (99,117), while good and incomplete occlusion decreased to 6.8% (8/117) and 8.6% (10/117), respectively (Table 2).

**Table 2:** The outcomes of treatment.

	**Rate**
**Angiographic outcomes**	
**Immediate surgery**	
Complete occlusion	60.1% (90/145)
Good occlusion	20.0% (29/145)
Incomplete occlusion	17.9% (26/145)
Latest follow-up^*^	
Complete occlusion	84.6% (99/117)
Good occlusion	6.8% (8/117)
Incomplete occlusion	8.6% (10/117)
**Complication**	
Endovascular stenosis	10.5% (14/133)
Injured side branches	6.8% (9/133)
**Clinical follow-up**	
Cerebral hemorrhage	3.0% (4/133)
Symptomatic ischemic infarct	4.5% (6/133)
Poor neurologic outcome (mCS ≥3)	2.3% (3/133)
Treatment-related mortality	0.8% (1/133)

* The ratio of occlusion degree was statistically different between immediate result and latest follow-up (*Z* = −3.900, *P* < 0.001).

### Clinical outcomes and complications

There was no parent artery occlusion in this cohort study. A total of 14 patients presented narrowing of the parent artery, and nine patients had side branch injuries after stent deployment. Finally, symptomatic cerebral infarction was caused by narrowing of the parent artery in six patients (4.5%). Periprocedural cerebral infarct (up to 7 days after the procedure) occurred in four patients (3.0%) and delayed cerebral infarct occurred in two patients (1.5%).

Two cases of acute hemorrhage happened during coil release, and one patient died of severe cerebral hemorrhage. Of the two cases with delayed hemorrhage, one occurred 2 days after treatment for a SAC middle cerebral artery (MCA) aneurysm, and the other occurred 3 days after treatment for a ruptured fusiform posterior inferior cerebellar artery (PICA) aneurysm *via* the left main stem (LMS; Hunt–Hess grade 1). Antiplatelet treatment was totally suspended when post-hemorrhage was found. Hemorrhage in the two patients remained stable, aspirin (100 mg/day) was used after 3 days, and clopidogrel (75 mg/day) was reused after a week. There was one case of death, but the rest of the hemorrhage cases were considered minor events and recovered before hospital discharge.

In this cohort study, the final permanent morbidity (mCS ≥3) and mortality were 2.8% (3/133) and 0.8% (1/133), respectively.

### Difference of aneurysm between SAC and LSM

According to treatment options, there were 82 and 63 aneurysms in the SAC group and in the LSM group, respectively (Table 3). All multiple aneurysms were treated with LSM [(χ^2^ = 35.580, *p* < 0.01). Ruptured aneurysms were likely to be treated with SAC (χ^2^ = 7.767, *p* = 0.005). There were more fusiform aneurysms (χ^2^ = 18.958, *p* < 0.01) and/or small-sized aneurysms (*Z* = −2.692, *p* = 0.007) in the LSM group.

**Table 3:** The clinical information between SAC and LSM.

	**SAC**	**LSM**	**Test value**	***P*** **-value**
Age (mean ± SD, years)	54.3 ± 11.7	53.4 ± 12.6	0.436^a^	0.664
Ruptured aneurysm (*n*, %)	19.5% (16/82)	7.9% (5/63)	3.855^b^	0.050
Recurrent aneurysm (*n*, %)	4.9% (4/82)	11.1% (7/63)	1.974^b^	0.160
Multiple aneurysms (*n*, %)	0	36.5% (23/63)	35.580^b^	*P* < 0.01
Perforator involving aneurysm	29.3% (24/82)	41.3% (26/63)	2.271^b^	0.132
**Aneurysm size**			−2.692^c^	0.007
Small (≤7 mm)	43	47		
Middle size (7.1–9.9 mm)	34	14		
Large (10–24.9 mm)	4	2		
Giant (≥25 mm)	1	0		
**Aneurysm shape**			18.958^b^	*P* < 0.01
Saccular	52 (52/82)	17 (17/63)		
Fusiform	30 (30/82)	46 (46/63)		
**Aneurysm location**			−1.443^c^	0.149
**Distal anterior aneurysm**				
ACA	14	11		
MCA	15	19		
**Posterior circulation**				
PCA	10	8		
PICA	4	3		
AICA	2	3		
VA	22	11		
BA	12	6		
BT	3	2		
**Embolic degree at last follow-up**			−1.596^c^	0.110
Complete occlusion	80.0% (52/65)	90.4% (47/52)		
Good occlusion	7.7% (5/65)	5.8% (3/52)		
Incomplete occlusion	12.3% (8/65)	3.8% (2/52)		
**Periprocedural complication**				
Parent artery narrowing	11.0% (9/82)	7.8% (4/51)	0.350^b^	0.554
Side branches injured	6.1% (5/82)	7.8% (4/51)	0.152^b^	0.697
Cerebral hemorrhage	3.7% (3/82)	2.0% (1/51)	0.311^b^	0.577
Symptomatic ischemic infarct	4.9% (4/82)	3.9% (2/51)	0.067^b^	0.796
Poor neurologic outcome	2.4% (2/82)	2.0% (1/51)	0.033^b^	0.857
Treatment-related mortality	1.2% (1/82)	0	0.627^b^	0.429

a T value;

b χ^2^ value;

c *Z* value; The normal distribution measurement data were showed by mean and standard deviation and the non-normal distribution measurement data were showed by median and quartile spacing. ACA, anterior cerebral artery; MCA, middle cerebral artery; PCA, posterior cerebral artery; PICA, posterior inferior cerebellar artery; AICA, anterior inferior cerebellar artery; VA, vertebral artery; BA, Basilar artery; BT, Basilar tips.

#### SAC group

There were 52 saccular and 30 fusiform aneurysms in the SAC group. After immediate surgery, the angiographic result showed complete occlusions (RROC 1), neck remnants (RROC 2), and residual aneurysms (RROC 3a) in 50.0% (41/82), 25.6% (21/82), and 24.4% (20/82), respectively. The degree of occlusion of the 65 aneurysms at the last follow-up showed 80.0% aneurysms in RROC 1, 7.7% in RROC 2, and 12.3% in RROC 3a. Two aneurysms with RROC 2 at immediate surgery showed neck recurrence during a follow-up and received EVT again.

In this group, intra-stent stenosis occurred in nine patients (11.0%) and injured side branches occurred in five cases (6.1%). In the early period after surgery, the rates of procedure-related complication rates of cerebral hemorrhage and symptomatic ischemic infarction were 3.7% (3/82) and 4.9% (4/82), respectively. Only one case of death in our cohort study was in the SAC group.

#### LSM group

There were 17 saccular aneurysms and 46 fusiform aneurysms in the LSM group. Of the 46 fusiform aneurysms, the average diameter was 6.5 ± 3.8 mm (2.48–22.9 mm); and according to Zhang's modified classification of fusiform aneurysms (12), 28 (60.9%, 28/46) aneurysms were classified as type I, 15 (32.6%) were type II, 3 (6.5%) were type III, and no aneurysms were type IV. After immediate surgery, imaging revealed OKM grade D in 49 aneurysms (77.8%), OKM grade C in eight aneurysms (12.7%), and OKM grade B in six aneurysms (9.5%). As shown in the last follow-up angiogram of 52 patients, the rate of complete occlusion (OKM grade D) was 90.4%. Five aneurysms remained stable (three aneurysms in OKM grade C and two aneurysms in OKM grade B), and no aneurysms worsened.

Of eight patients with intra-stent stenosis or injured side branches, two developed symptomatic ischemic infarction. One patient with a ruptured fusiform aneurysm in PICA suffered a recurrence of cerebral hemorrhage 2 days after the deployment of a Leo plus stent and totally recovered with conservative treatment. At prolonged follow-up, morbidity was 1.9% (1/62).

## Discussion

Endovascular treatment of aneurysms in the posterior and distal anterior circulations remains a challenge for interventional neuroradiologists. Delivery of conventional stiff flow-diverting stents in small or tortuous arteries would be dangerous, and retrieval of the system could be challenging (13, 14). Leo, as an existing self-expandable stent, has been proven to have FD properties (5, 9). To the best of our knowledge, our cohort is the biggest to study the FD effect of Leo stents for such aneurysms and to analyze treatment strategies for different characteristic aneurysms.

### Occlusion results

As the final angiographic results of our study showed that the rate of complete occlusion (RROC 1) was 80.0% in the SAC group, which was similar to the main results of the last 10 years (from 70 to 88.9%) (15–21). In the LSM group, 90.3% of aneurysms in complete occlusion (OKM grade D) was the highest rate compared to the results from other LSM studies [70.0% (9), 73.7% (5)], even higher than TuBridge flow diverters (50% in OKM grades D and C) (22) or a meta-analysis of flow-diverting stents (78.7% in complete/near complete occlusion) (23) in MCA. For complicated aneurysms, the two-stage operation is accepted due to the high risk of rupture during coiling (24).

### FD of leo stents

**Figure 1:**
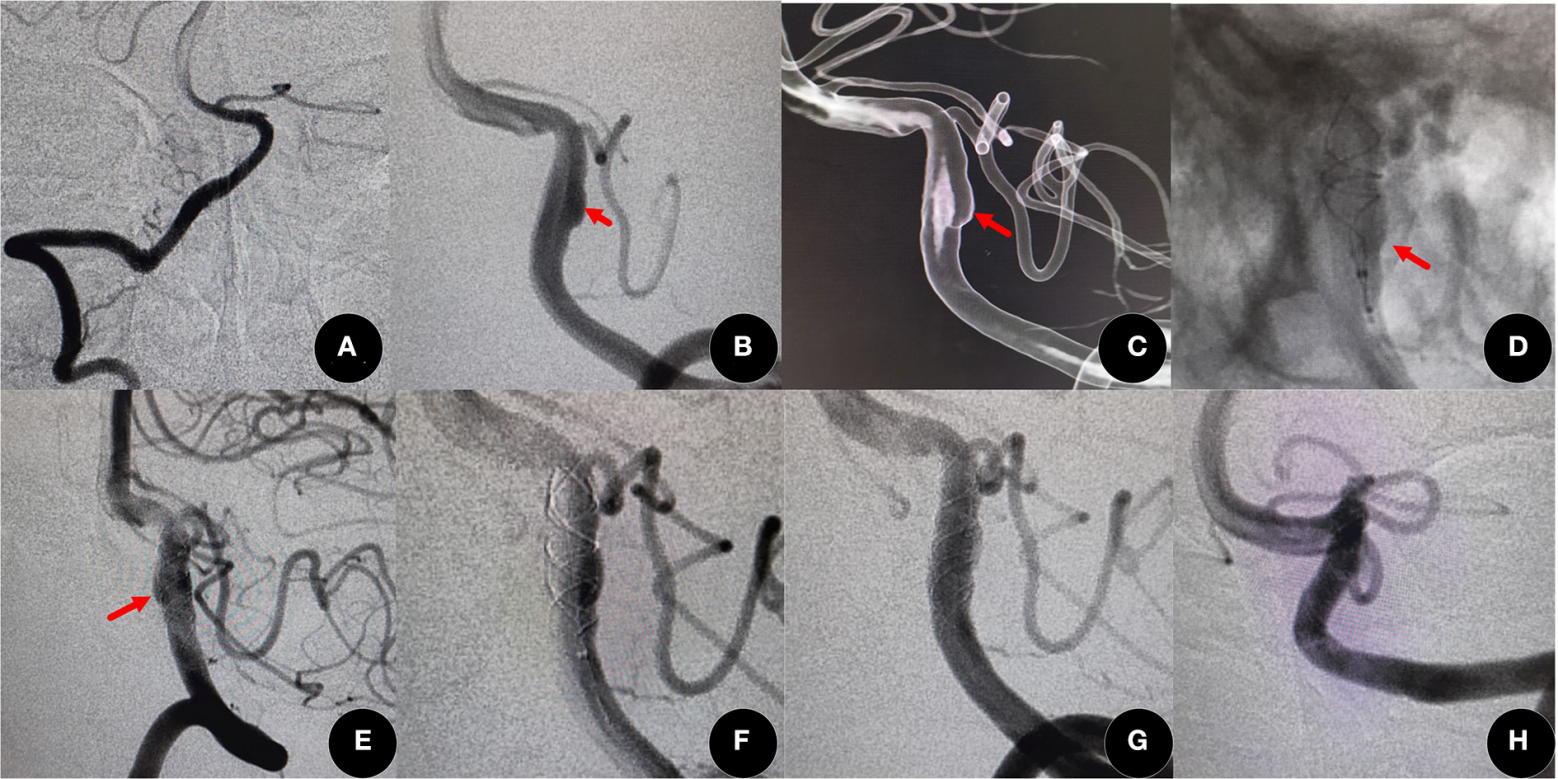
On routine examination, a fusiform aneurysm was found (60-year-old women). **(A)** Normal right vertebral artery. **(B,C)** Angiographic images showed a left fusiform vertebral artery (red arrow) **(D,E)** A Leo plus stent (3.5 mm × 25 mm) was deployed by telescopic technique (red arrow), and the aneurysm incomplete occlusion [O'Kelly Marotta (OKM) grade B]. **(F,G)** After the deployment of another Leo plus stent of the same size, flow into the aneurysm was obviously decreased and the degree of occlusion improved to good occlusion (OKM grade C). **(H)** The degree increased to complete occlusion (OKM grade D) at 14 months after surgery.

**Figure 2:**
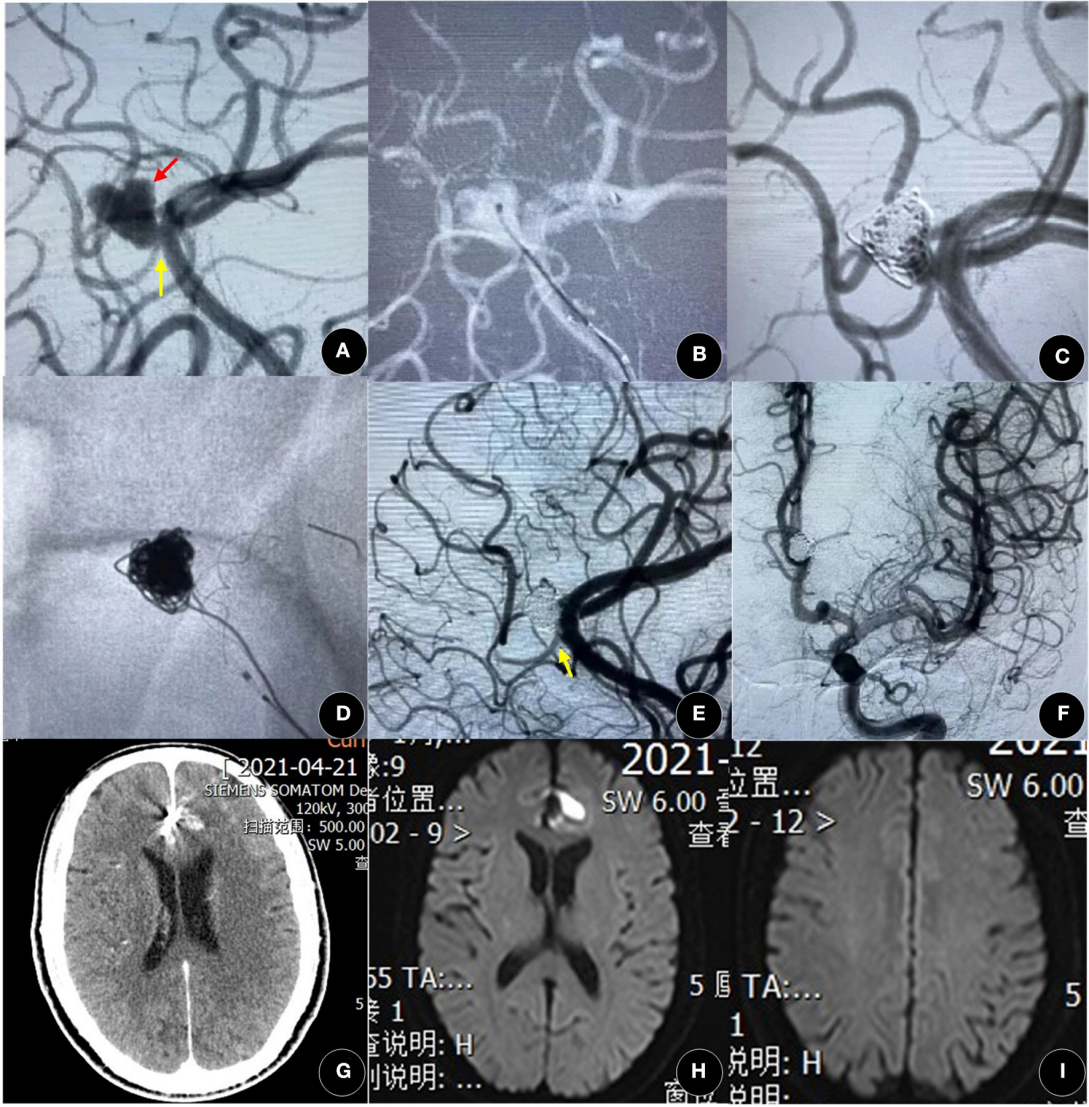
A 60-year-old woman presented with spontaneous SAH for 3 days. **(A)** The angiographic result showed a left lobular A3 aneurysm (black arrow) with a side branch (yellow arrow). **(B,C)** After coiling, the aneurysm was in incomplete occlusion [Raymond-Ray Occlusion Class (RROC) 3c]. **(D–F)** The aneurysm was achieved in complete occlusion (RROC 1) after the deployment of the Leo plus baby stent (2.5 mm × 18 mm), and the side branch was not affected (yellow arrow). **(G–I)** There was no new cerebral hemorrhage or infarction by computed tomography (CT) and magnetic resonance imaging (MRI). A3, the third segment of the anterior cerebral artery; SAH, subarachnoid hemorrhage.

**Figure 3:**
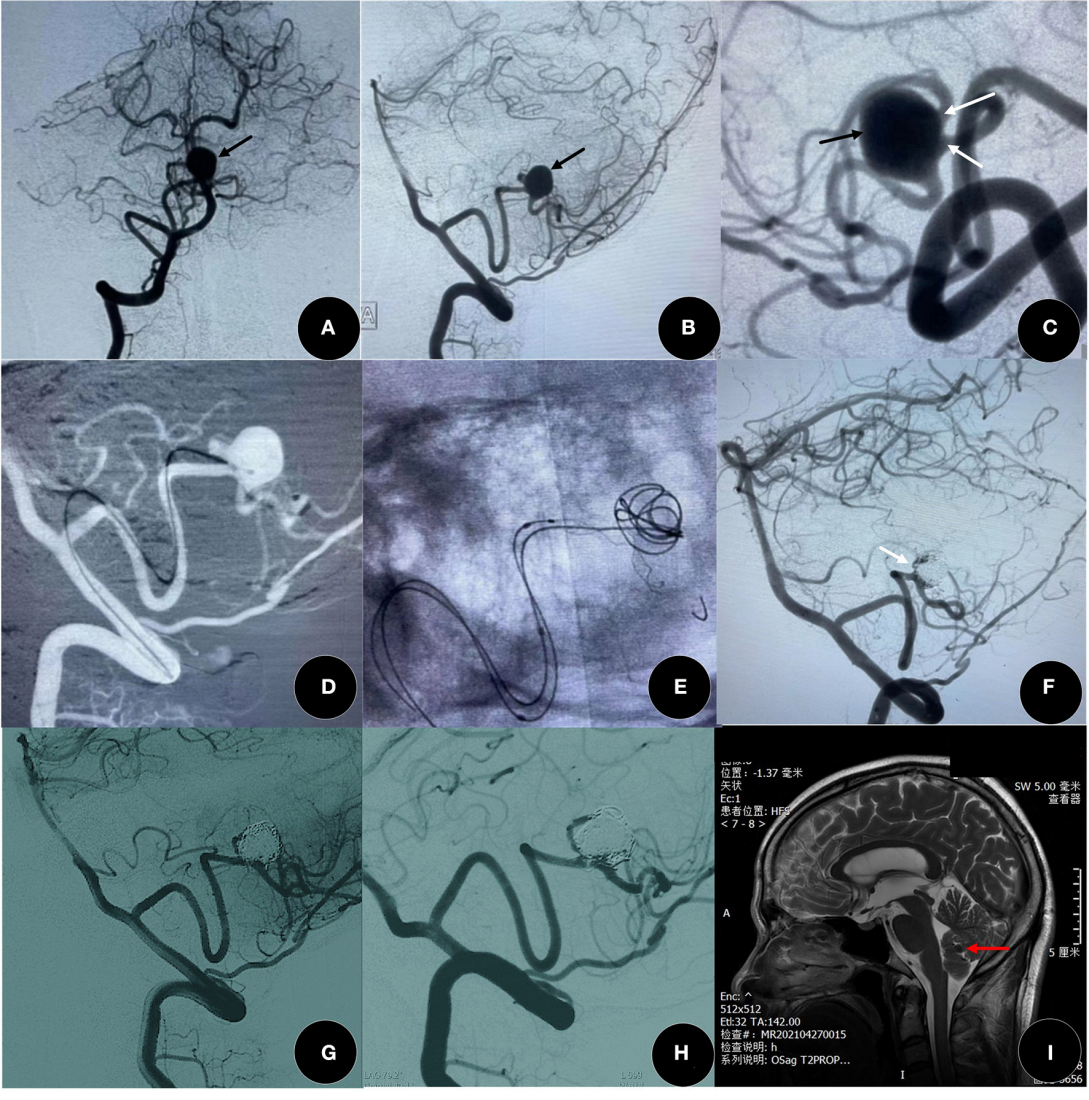
A 51-year-old woman presented with a headache for 1 year. **(A–C)** The angiographic result showed a distal posterior inferior cerebellar artery (PICA) aneurysm (black arrow) with two side branches (white arrow). **(D–G)** The aneurysm was in good occlusion (RROC 2) by Leo plus baby (2.5 mm × 18 mm) assisted coiling, and two branches were well-protected (white arrow). **(G,H)** The degree of occlusion increased to complete occlusion (RROC 2) at 9 months after surgery. **(I)** There is no new cerebral infarction after the confirmation of SAC by MRI, and red arrow indicates coils. SAC, stent-assisted coiling.

Pumar et al. found that the rate of complete occlusion (RROC 1) of wide-neck intracranial aneurysms after Leo stent deployment increased from 39.8% at immediate post-procedural angiography to 73.1% at year 5 (15). In our study, the degree of angiographic occlusion had improved significantly during a follow-up, similar to other studies (16, 25), again demonstrating that Leo stents have the ability to persistently promote thrombosis in the aneurysm due to the FD effect (Figure 1). Based on our clinical experience, overlapping stents (26) and telescopic technique (5) are also two important practical techniques of Leo stents to increase mesh density and improve the FD effect (Figure 2). Leo stents with favorable elasticity can easily be released and opened in small and tortuous distal parent arteries (Figure 3).

### Procedure-related complications

Flow-diverting stents with high metal coverage may easily cause occlusion of the parent artery and injured side branches (2, 5, 13, 27, 28). A meta-analysis showed that more than 10% of covered arteries became occluded during a follow-up, with ~16.3% thromboembolic events due to flow-diverting stents in MCA aneurysms (23). In this study, of 14 patients with parent artery narrowing or side branch lesions, four had developed a cerebral infarction.

Many related risks associated with the development of ischemia have been studied. Matteau et al. found that smoking was an independent risk factor for ischemic events and bleeding after stent implantation (29). Cagnazzo et al. showed that the rates of arterial narrowing and occlusion by Leo were close to 7 and 2%, respectively, and a longer radiologic follow-up and smoking are two independent factors associated with arterial narrowing and occlusion (9).

Delayed aneurysm rupture and distal intraparenchymal hemorrhage were the main causes of mortality in patients with intracranial aneurysms treated with flow-diverting stents (30). In our cohort, four patients had procedure-related hemorrhage complications, of which two were caused by coiling, and one died at 2 weeks even after lateral ventricular drainage. Two patients suffered delayed hemorrhage, we will totally stop antiplatelet treatment for 3 days, if the hemorrhage stops and remains stable, monoclonal antibody will be used and if hemorrhage remains stable for a week, the double resistance will be reused. Although the incidence is not high, postoperative hemorrhage was still a nightmare for interventional neuroradiologists. In our study, most of the procedure-related complications had mild to moderate neurological deficits and disappeared within 3 months and we did not find any potential risk factors with procedure-related complications. Popularly, the intensity of antiplatelet therapy was decreased at 3 months after surgery (from 2 to 1) (17, 18) and terminated at 2 years after surgery.

### Therapeutic strategy

**Figure 4:**
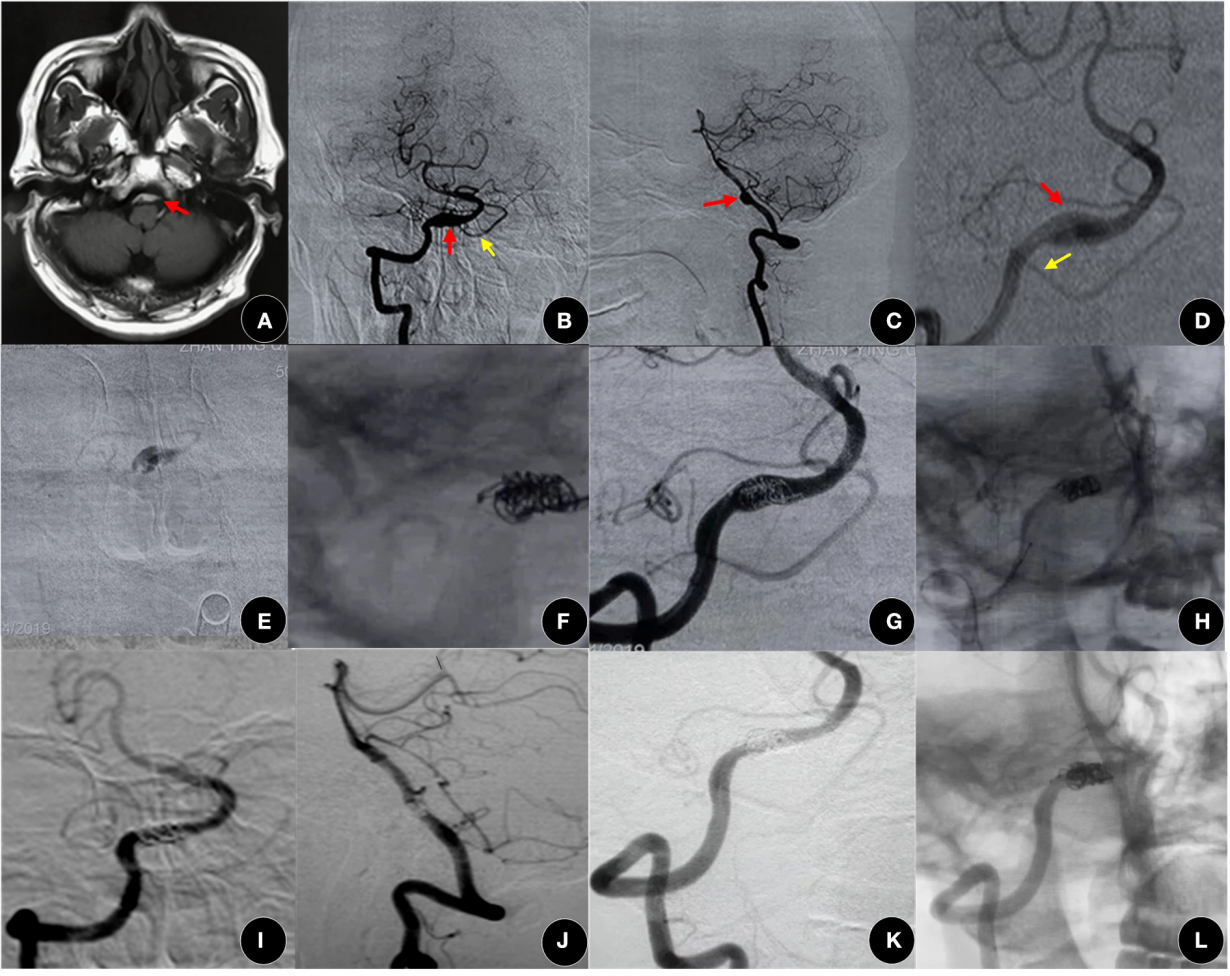
A 53-year-old man presented with SAH for 2 days. **(A–D)** MRI images indicated an aneurysm in the vertebrobasilar system (red arrow). **(B–D)** Angiographic images showed a vertebral artery dissecting aneurysm (red arrow) with a double aneurysmal cavity, located in the proximal posterior inferior cerebellar artery (yellow arrow). **(E)** It was confirmed that the microcatheter was in the aneurysmal cavity by the hand bolus injection contrast medium. **(F)** After releasing several coils, a Leo plus stent (3.5 mm × 25 mm) was deployed to press these coils evenly into the cavity, and then the other coils were continuously packed. **(G,H)** Good occlusion (OKM grade C) was achieved, and the VA and PICA were patented. **(I–L)** The embolism degree increased to complete occlusion (OKM grade D) and the modified Rankin scale (mRS) maintained a score of 0 at 13 months after surgery. VA, vertebral artery; PICA, posterior inferior cerebellar artery.

Although there was no significant difference in the degree of occlusion and postoperative complications, we proposed that SAC was still a priority, especially for irregularly shaped aneurysms (Figure 2) or ruptured aneurysms (Figure 4). Intraoperative aneurysm rupture is one of the significant risk factors for early serious complications (17), and the risk of intraprocedural rupture is significantly high during coiling, especially in heteromorphic or very small aneurysms (31, 32). Flow velocity in the aneurysm and wall shear stress are two important hemodynamic parameters associated with the growth and rupture of intracranial aneurysms (7, 8), and could be eliminated by coiling the aneurysm more intuitively (33, 34). In our study, two cases with acute cerebral hemorrhage were all in the SAC group, in our experience, complicated aneurysms treated with Leo-assisted coiling could not be packed too densely.

In 2008, a fusiform M_1_ MCA aneurysm was satisfactorily treated with LSM, demonstrating that the Leo stent could cause flow reduction and final thrombosis in the aneurysm without any additional treatment (35). Even without the expected occlusion, the fusiform aneurysms with Leo monotherapy could remain clinically and angiographically stable during a follow-up (36). As per our experience, sometimes the coil microcatheter would be difficult to introduce into the aneurysm located in the distal and tortuous artery, LSM was also demonstrated to be an important proposed alternative for saccular aneurysms and the incidence of intraprocedural rupture would decrease markedly without catheterization or a coil in the aneurysm. As we consider that small distal parent arteries have difficulty supporting three or more microcatheters simultaneously, many aneurysms are already achieved in complete occlusion after Leo stent deployment.

## Limitations

The sample size in our cohort here was still not large. Of 133 patients, 21 (15.8%) had not received angiographic follow-up, which might lead to an attrition bias, and the follow-up was not long. In the future, as the number of cases increases, aneurysms located in the posterior and the distal anterior circulations should be analyzed individually with longitudinal follow-up, and a prospective multicenter study may be performed.

## Conclusion

Leo stents are safe and effective for aneurysms located in the posterior and distal anterior circulations. The degree of occlusion of aneurysms had persistently improved during a follow-up due to the FD effect of Leo stents. According to the different characteristics, those complicated aneurysms should be treated with personalized measures.

## Data availability statement

The raw data supporting the conclusions of this article will be made available by the authors, without undue reservation.

## Ethics statement

The studies involving human participants were reviewed and approved by the Institutional Review Board of Huashan Hospital and Huadong Hospital, Shanghai Putuo District People's Hospital. Written informed consent to participate in this study was provided by the participants' legal guardian/next of kin. Written informed consent was obtained from the individual(s) for the publication of any potentially identifiable images or data included in this article.

## Author contributions

GC designed the study and performed surgery. YD wrote the article and analyzed the data. BX, RM, XQ, and JL assisted to finish part of surgery. YH and BZ collected the data. All authors contributed to the article and approved the submitted version.

## Funding

This work was funded by Project of Famous Medical Doctors of Shanghai Medical College of Fudan University (DGF828007/028) and Shanghai Science and Technology Committee (No. 18411962400).

## Conflict of interest

The authors declare that the research was conducted in the absence of any commercial or financial relationships that could be construed as a potential conflict of interest.

## Publisher's note

All claims expressed in this article are solely those of the authors and do not necessarily represent those of their affiliated organizations, or those of the publisher, the editors and the reviewers. Any product that may be evaluated in this article, or claim that may be made by its manufacturer, is not guaranteed or endorsed by the publisher.
